# Physiologic Preoperative Knee Hyperextension Is Not Associated With Postoperative Laxity, Subjective Knee Function, or Revision Surgery After ACL Reconstruction With Hamstring Tendon Autografts

**DOI:** 10.1177/03635465241288238

**Published:** 2024-10-23

**Authors:** Gunnar Edman, Kristian Samuelsson, Eric Hamrin Senorski, Romain Seil, Riccardo Cristiani

**Affiliations:** * Research and Development, Norrtälje Hospital, Tiohundra AB, Norrtälje, Sweden; † Department of Clinical Sciences, Danderyd Hospital, Karolinska Institutet, Stockholm, Sweden; ‡ Department of Orthopaedics, Institute of Clinical Sciences, Sahlgrenska Academy, University of Gothenburg, Gothenburg, Sweden; § Sports Clinic, Centre Hospitalier de Luxembourg-Clinique d’Eich, Luxembourg, Luxembourg; ‖ Luxembourg Institute of Research in Orthopaedics, Sports Medicine and Science, Luxembourg, Luxembourg; ¶ Human Motion, Orthopaedics, Sports Medicine and Digital Methods, Luxembourg Institute of Health, Strassen, Luxembourg; # Department of Molecular Medicine and Surgery, Stockholm Sports Trauma Research Center, Karolinska Institutet, Stockholm, Sweden; ** Capio Artro Clinic, FIFA Medical Centre of Excellence, Stockholm, Sweden; Investigation performed at Stockholm Sports Trauma Research Center, Karolinska Institutet, Stockholm, Sweden

**Keywords:** ACL reconstruction, hyperextension, knee laxity, patient-reported outcome, revision ACL reconstruction

## Abstract

**Background:**

There is concern that physiologic knee hyperextension may be associated with inferior outcomes after anterior cruciate ligament reconstruction (ACLR) using hamstring tendon (HT) autografts.

**Purpose:**

To assess whether there is an association between contralateral passive preoperative knee hyperextension (≤−5°) and postoperative anterior knee laxity, subjective knee function, or revision surgery after ACLR using HT autografts.

**Study Design:**

Cohort study; Level of evidence, 3.

**Methods:**

Patients without concomitant ligament injuries who underwent primary ACLR using an HT autograft at Capio Artro Clinic, Stockholm, Sweden, between January 1, 2005, and December 31, 2018, were identified. The cohort was dichotomized into the hyperextension group (≤−5°) and the no hyperextension group (>–5°) depending on preoperative contralateral passive knee extension degree. Anterior knee laxity (KT-1000 arthrometer; 134 N) was assessed preoperatively and at 6 months postoperatively. The Knee injury and Osteoarthritis Outcome Score (KOOS) was collected preoperatively and at 1, 2, and 5 years postoperatively. Patients who underwent revision ACLR at any institution in Sweden within 5 years of the primary surgery were identified in the Swedish National Knee Ligament Registry.

**Results:**

A total of 6104 patients (53.5% male) for whom knee range of motion measurements were available were identified (hyperextension group [≤−5°]: 2350 [38.5%]; mean extension, −6.1°± 2.3° [range, −20° to −5°]; no hyperextension group [>−5°]: 3754 [61.5%]; mean extension, 0°± 1.4° [range, −4° to 15°]). There were no intergroup differences in anterior knee laxity preoperatively (hyperextension group, 3.6 ± 2.8 mm; no hyperextension group, 3.7 ± 2.7 mm; *P* = .24) or postoperatively (hyperextension group, 1.8 ± 2.3 mm; no hyperextension group, 1.8 ± 2.2 mm; *P* = .41). The only significant but nonclinically relevant intergroup differences were seen in the KOOS Symptoms subscale at the 1-year follow-up (hyperextension group, 81.4 ± 16.0; no hyperextension group, 80.3 ± 16.5; *P* = .03) and in the Sport and Recreation subscale at the 5-year follow-up (hyperextension group, 73.0 ± 25.6; no hyperextension group, 75.7 ± 24.3; *P* = .02). No other significant intergroup differences were noted preoperatively or at 1, 2, or 5 years postoperatively in any of the KOOS subscales. The overall revision ACLR rate at ≤5 years after the primary surgery was 4.9% (302 of 6104 patients). The hazard for revision ACLR in the no hyperextension group (4.5%; 170 of 3754 patients) was not significantly different from that in the hyperextension group (5.6%; 132 of 2350 patients) (hazard ratio, 0.89; 95% CI, 0.71 to −1.12; *P* = .34). A subsequent subanalysis showed that the hazard of revision ACLR in patients with no hyperextension was not significantly different from that of patients with ≤−10° of extension (5.8%; 27 of 467 patients) (hazard ratio, 0.91; 95% CI, 0.61 to 1.36; *P* = .65).

**Conclusion:**

Preoperative passive contralateral knee hyperextension (≤−5°) was not associated with postoperative anterior knee laxity, subjective knee function, or revision surgery ≤5 years after ACLR using HT autografts. Therefore, the presence of knee hyperextension alone should not be considered a contraindication per se for the use of HT autografts in ACLR.

Anterior cruciate ligament (ACL) reconstruction (ACLR) is able to restore knee laxity and improve subjective knee function, and it is associated with an overall low revision rate.^4-6^ However, many factors may affect surgical results. Generalized joint hypermobility is consistently associated with inferior ACLR outcomes. In a recent systematic review, Sundemo et al^32^ reported that generalized joint hypermobility was associated with greater postoperative knee laxity and inferior patient-reported outcomes. Larson et al^20^ and Helito et al^15^ reported failure rates of >20% (24.4% and 21.7%, respectively) after ACLR in patients with generalized joint hypermobility versus approximately 6% in the general population.^38^ However, clinical studies evaluating the effects of knee hyperextension alone on ACLR outcomes are scarce. Biomechanical studies have suggested that knee hyperextension has deleterious effects on the ACL. Markolf et al^22,23^ found higher ACL forces during knee hyperextension. Similarly, Jagodzinski et al^17^ reported that full passive knee extension exerted biomechanical pressure and tension on the ACL, with the highest forces achieved during passive knee hyperextension. Despite this biomechanical evidence, it is unclear whether knee hyperextension affects ACLR outcomes. The few existing studies have yielded mixed results. In a cohort of 625 patients who underwent single-bundle ACLR with a bone-patellar tendon-bone (BPTB) autograft, Benner et al^1^ found that knee hyperextension did not affect postoperative knee laxity, ACL graft tear/failure rates, or subjective knee function. In contrast, in a matched group analysis of 204 patients (102 with knee hyperextension and 102 without knee hyperextension) who underwent single-bundle ACLR using hamstring tendons (HTs), Guimarães et al^12^ found that patients with knee hyperextension reported inferior subjective knee function and had 5 times higher failure rates compared with patients without knee hyperextension. However, to date, these findings have not been confirmed or refuted by other authors, and there is a lack of large-cohort registry studies evaluating the effects of knee hyperextension on postoperative knee laxity, subjective knee function, and the risk of revision surgery after primary ACLR using HT autografts.

This study aimed to assess whether preoperative contralateral passive knee hyperextension (≤−5°) is associated with postoperative anterior knee laxity, subjective knee function, or revision surgery after ACLR using HT autografts in a large cohort of patients. We hypothesized that patients with contralateral passive knee hyperextension (≤−5°) would have increased knee laxity, inferior subjective knee function, and an increased hazard of revision ACLR compared with patients with no hyperextension (>−5°).

## Methods

### Participants

Patients without concomitant ligament injuries who underwent primary ACLR using an HT autograft at Capio Artro Clinic, Stockholm, Sweden, between January 1, 2005, and December 31, 2018, were assessed for eligibility. Patients with contralateral ACL injury or reconstruction or missing preoperative range of motion (ROM) measurements were excluded.

### Surgical Technique and Rehabilitation

A single-bundle technique was used for all ACLRs. The semitendinosus tendon was primarily harvested and prepared as a tripled or quadrupled graft. In the event of insufficient length or diameter (<8 mm), the gracilis tendon was harvested and combined with the semitendinosus tendon graft. An anteromedial portal technique was used to drill the femoral tunnel. The graft was routinely fixed using an Endobutton (Smith & Nephew) or Tightrope (Arthrex) fixation device on the femoral side and Ethibond No. 2 sutures (Ethicon) tied over an AO bicortical screw with a washer as a post or with an interference screw on the tibial side. An arthroscopic all-inside technique using Fast-Fix suture anchor devices (Smith & Nephew) was used to repair meniscal tears located in the posterior horn and corpus of the menisci. Meniscal tears located in the anterior portion of the menisci were repaired via an outside-in technique using PDS 0 (Ethicon). The patients followed a supervised and standardized postoperative rehabilitation protocol. Full weightbearing and ROM were allowed in the event of an isolated ACLR or ACLR and concomitant meniscal resection. In the event of an ACLR and concomitant meniscal repair, the patients wore a hinged knee brace for 6 weeks. The brace was set to allow for 0° to 30° of flexion for the first 2 weeks, 0° to 60° of flexion for the third and fourth weeks, and 0° to 90° for the fifth and sixth weeks postoperatively. Starting from the seventh week, brace use was discontinued, and full ROM was encouraged. Quadriceps strengthening was restricted to closed kinetic chain exercises during the first 3 months. The patients were allowed to return to sports 6 months after ACLR at the earliest if the isokinetic knee muscle strength and single-leg hop (SLH) test criteria [limb symmetry index ≥90%] were met.^7,36^

### Range of Motion Measurements

**Figure 1.**
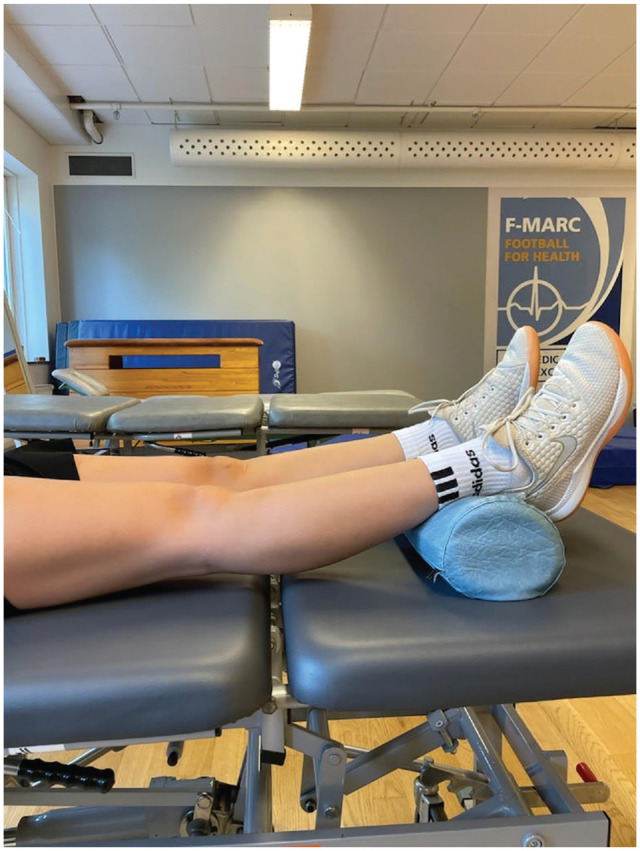
The patient's heels were placed on a bolster.

Passive ROM was measured preoperatively using a goniometer. The patient was supine, and to assess knee hyperextension, we placed the patient's heels on a bolster (Figure 1). The goniometer was centered over the lateral femoral epicondyle. The proximal arm was aligned with the lateral midline of the femur using the greater trochanter as reference, whereas the distal arm was aligned with the lateral midline of the fibula, using the lateral malleolus as reference.^25^ All measurements were performed bilaterally by experienced sports medicine physical therapists at our outpatient clinic. For the purposes of this study, passive contralateral knee extension was used to avoid the effects of the ACL injury on the injured knee. The cohort was dichotomized into the hyperextension group (≤−5°) and the no hyperextension group (>−5°) depending on the degree of knee extension.

### Isokinetic Muscle Strength and SLH Test

Muscle strength and the SLH test were assessed 6 months postoperatively. Isokinetic concentric extension and flexion strength were measured using the Biodex System 3 (Biodex Medical Systems). An angular velocity of 90 deg/s was used, and all measurements were performed bilaterally in an ROM between 90° and 10°. Patients warmed up using a stationary cycling ergometer at low resistance for about 10 minutes before the test. Oral instructions about the test were given, and 2 to 3 practical trials were allowed before the test. Five maximum repetitions (extension and flexion) were performed bilaterally, and the peak torque values (highest values achieved) were registered. The SLH test was also performed.^27^ Instructions were given to stay on 1 leg, jump straight ahead as far as possible, and land on the same leg. If the landing was stable, the test was considered successful. In cases of landing with an early touchdown of the opposite leg, additional hops after landing, or loss of balance, the hop was repeated. Oral instructions about the test were given, and several practical trials were allowed. The contralateral, uninjured leg was tested first, followed by the injured leg. Three trials for each leg were performed, and the best trial was registered.^4,6,7^

### Arthrometric Knee Laxity

Knee laxity was assessed preoperatively and 6 months postoperatively using the KT-1000 arthrometer (MEDmetric). A standard anterior tibial load of 30 lb (134 N) was used, and the measurements were performed at 20° of knee flexion. All assessments were performed by experienced sports medicine physical therapists at our outpatient clinic. At least 3 measurements of each knee were performed, and the median value was registered. The side-to-side (STS; injured knee/healthy knee) difference was registered. STS laxity was then stratified into 3 groups (≤2 mm, 3-5 mm, and >5 mm) according to the International Knee Documentation Committee (IKDC) examination form.^13^

### Subjective Knee Function

Subjective knee function was evaluated preoperatively and at 1, 2, and 5 years postoperatively using the Knee injury and Osteoarthritis Outcome Score (KOOS).^29,30^ The KOOS consists of 5 subscales: Symptoms, Pain, Activities of Daily Living, Sport and Recreation (Sport/Rec), and knee-related Quality of Life. The scores are reported as a number for each subscale, with 0 representing the worst possible outcome and 100, the maximum score.

### Revision ACLR

Patients who underwent revision ACLR ≤5 years after primary surgery at any institution in Sweden between January 1, 2005, and December 31, 2023, were identified using their unique Swedish personal identity number,^21^ in the Swedish National Knee Ligament Registry (SNKLR; http://www.aclregister.nu).

### Data Sources

Data collected from our clinic database were preoperative contralateral passive knee ROM, age at primary ACLR, time from injury to primary ACLR, sex, the presence of a cartilage injury, meniscal injury (medial or lateral), meniscal surgery (classified as resection or repair of the medial meniscus or lateral meniscus [LM]), preinjury Tegner activity level,^35^ graft diameter, 6-month isokinetic (extension and flexion) strength, and SLH test performance. Preoperative and 6-month postoperative KT-1000 arthrometric laxity measurements were also collected from our clinic database. The KOOS subscale scores were extracted from the SNKLR.

### Statistical Analysis

The Statistical Package for Social Sciences (Version 25.0; IBM Corp) was used for the statistical analysis. The variables were summarized using standard descriptive statistics, such as the mean, standard deviation, or frequency. The distributions were checked for deviation from a normal distribution. Independent-samples Student *t* test and Pearson chi-square test were used for comparisons of continuous and categorical variables, respectively. Mean preoperative and postoperative knee laxity values were analyzed using analysis of covariance with age at surgery and sex as covariates.^5^ Differences in preoperative and postoperative STS laxity according to the IKDC examination form^13^ were analyzed using the Pearson chi-square for trend. Preoperative (covariates: age at surgery, sex, cartilage injury, and LM injury) and 1-, 2-, and 5-year postoperative (covariates: age at surgery, sex, cartilage injury, and LM repair) KOOS subscale scores were compared using analysis of covariance.^3,6,11^ A Cox regression analysis was used to compare the 5-year revision risk between the hyperextension group and the no hyperextension group. Hazard ratios (HRs) were adjusted for age at surgery, sex, cartilage injury, and LM repair. These potential confounders were added to the model because there were differences between the groups and previous studies have shown that these factors may potentially affect the risk of revision ACLR.^4,28,33^ The level of significance in all analyses was 5% (2-tailed).

## Results

A total of 7206 patients were reviewed for eligibility. After excluding patients with a contralateral ACL injury or reconstruction (n = 518) and patients with no available contralateral ROM measurements (n = 584), 6104 patients were included. The included patients were dichotomized into the hyperextension group (≤−5°) and the no hyperextension group (>−5°) depending on preoperative contralateral passive knee extension degree. The patient characteristics are summarized in Table 1.

**Table 1:** Patient Characteristics^*a*^

	No Hyperextension (>−5°)	Hyperextension (≤−5°)	*P*
Patients	3754 (61.5)	2350 (38.5)	
Knee extension, mean ± SD (range), deg	00 ± 1.4 (−4 to 15)	−6.1 ± 2.3 (−20 to −5)	<.001^*b*^
Age at surgery, mean ± SD, y	29.1 ± 11.0	27.1 ± 10.5	<.001^*b*^
Time from injury to surgery, mean ± SD, mo	20.2 ± 41.5	19.6 ± 38.5	.61
Patients with available data, n	3713	2313	
Sex			<.001^*b*^
Male	2087 (55.6)	1176 (50.0)	
Female	1667 (44.4)	1174 (50.0)	
Cartilage injury	702 (18.7)	376 (16.0)	.007^*b*^
MM injury	852 (22.7)	571 (24.3)	.15
LM injury	998 (26.6)	555 (23.6)	.01^*b*^
MM resection	587 (15.6)	337 (14.3)	.17
MM repair	306 (8.1)	172 (7.3)	.77
LM resection	587 (15.6)	374 (15.9)	.23
LM repair	163 (4.3)	129 (5.5)	.04^*b*^
Preinjury Tegner score, mean ± SD	7.1 ± 1.7	7.3 ± 1.7	.16
Patients with available data, n	3543	2265	
Graft diameter, mean ± SD, mm	8.2 ± 1.5	8.1 ± 1.5	.54
Patients with available data, n	3105	1868	
6-mo isokinetic extension strength, LSI, mean ± SD	84.9 ± 16.3	85.5 ± 15.3	.20
Patients with available data, n	3109	2000	
6-mo isokinetic flexion strength, LSI, mean ± SD	89.8 ± 21.2	89.5 ± 14.7	.47
Patients with available data, n	3107	1994	
6-mo SLH test performance, LSI, mean ± SD	93.1 ± 12.8	93.0 ± 12.4	.62
Patients with available data, n	2561	1710	

a Data are reported as n (%) unless otherwise indicated. LM, lateral meniscus; LSI, limb symmetry index; MM, medial meniscus; SLH, single-leg hop. n, number.

b Statistically significant.

### Arthrometric Knee Laxity

There were no differences between the groups (preoperative and postoperative) either in mean STS laxity or in the STS laxity values stratified according to the IKDC knee examination form^13^ (Table 2).

**Table 2:** Mean and Stratified KT-1000 Arthrometer Side-to-Side Difference Values^*a*^

	No Hyperextension (>−5°)	Hyperextension (≤−5°)	*P*
Preoperative STS mean ± SD, mm	3.7 ± 2.7	3.6 ± 2.8	.24
≤2	1072 (33.1)	692 (33.1)	.11
3-5	1405 (43.4)	955 (45.7)	
>5	758 (23.4)	442 (21.2)	
Patients with available data, n	3235	2089	
Postoperative STS, mean ± SD, mm	1.8 ± 2.2	1.8 ± 2.3	.41
≤2	1716 (63.3)	1124 (63.3)	.42
3-5	855 (31.5)	574 (32.3)	
>5	140 (5.2)	77 (4.3)	
Patients with available data, n	2711	1775	

a Data are reported as n (%) unless otherwise indicated. STS, side-to-side.

### Subjective Knee Function

The only significant differences between the groups were seen in the KOOS Symptoms subscale at the 1-year follow-up (hyperextension group, 81.4 ± 16.0; no hyperextension group, 80.3 ± 16.5; *P* = .03) and in the Sport/Rec subscale at the 5-year follow-up (hyperextension group, 73.0 ± 25.6; no hyperextension group, 75.7 ± 24.3; *P* = .02). No other significant differences between the groups were found preoperatively or at 1, 2, or 5 years postoperatively for any of the KOOS subscales (Table 3).

**Table 3:** Preoperative and Postoperative KOOS Comparison^*a*^

	No Hyperextension (>−5°)	Hyperextension (≤−5°)	*P*
Symptoms			
Preoperatively	3748 (75.6 ± 17.6)	2346 (76.2 ± 17.8)	.28
1 y	2803 (80.3 ± 16.5)	1746 (81.4 ± 16.0)	.03^*b*^
2 y	1990 (82.6 ± 15.7)	1299 (82.2 ± 15.9)	.89
5 y	1600 (84.6 ± 15.8)	1037 (83.1 ± 16.8)	.08
Pain			
Preoperatively	3746 (79.6 ± 16.2)	2346 (80.2 ± 15.9)	.35
1 y	2803 (87.8 ± 13.1)	1746 (88.2 ± 12.4)	.40
2 y	1990 (88.6 ± 13.2)	1299 (88.2 ± 13.7)	.59
5 y	1600 (89.4 ± 13.6)	1037 (88.3 ± 14.5)	.16
ADL			
Preoperatively	3745 (87.4 ± 14.5)	2345 (88.6 ± 14.0)	.06
1 y	2804 (94.1 ± 10.0)	1745 (94.5 ± 9.7)	.36
2 y	1990 (94.2 ± 10.4)	1299 (93.8 ± 11.4)	.22
5 y	1600 (94.3 ± 10.5)	1037 (93.9 ± 11.4)	.22
Sport/Rec			
Preoperatively	3541 (51.4 ± 27.9)	2226 (53.2 ± 27.1)	.09
1 y	2769 (72.1 ± 23.8)	1730 (72.0 ± 23.2)	.41
2 y	1964 (74.2 ± 23.2)	1289 (73.8 ± 24.2)	.72
5 y	1581 (75.7 ± 24.3)	1020 (73.0 ± 25.6)	.02^*b*^
QOL			
Preoperatively	3643 (40.0 ± 23.6)	2291 (40.6 ± 23.8)	.57
1 y	2791 (62.7 ± 22.9)	1735 (63.2 ± 22.8)	.55
2 y	1980 (67.3 ± 22.6)	1289 (67.2 ± 22.9)	.79
5 y	1588 (70.5 ± 23.6)	1030 (68.2 ± 24.2)	.08

a Data are reported as n (mean ± SD). ADL, Activities of Daily Living; KOOS, Knee injury and Osteoarthritis Outcome Score; QOL, Quality of Life; Sport/Rec, Sport and Recreation.

b Statistically significant.

### Revision ACLR

The overall revision ACLR rate within 5 years of primary surgery was 4.9% (302 of 6104 patients). The hazard of revision ACLR in the no hyperextension group (>−5°) (4.5%; 170 of 3754 patients) was not significantly different from that in the hyperextension group (≤−5°) (5.6%; 132 of 2350 patients) (HR, 0.89; 95% CI [95% CI], 0.71-1.12; *P* = .34).

Further subanalysis of the hyperextension group showed that the hazard of revision ACLR in patients with no hyperextension was not significantly different from that of patients with ≤−10° of extension (5.8%; 27 of 467 patients) (HR, 0.91; 95% CI, 0.61-1.36; *P* = .65).

## Discussion

The most important finding of the present study was that physiologic preoperative contralateral passive knee hyperextension (≤−5°) was not associated with postoperative anterior knee laxity, subjective knee function, or revision surgery ≤5 years after ACLR using HT autografts. Furthermore, a subanalysis of the hyperextension group showed that the hazard of revision ACLR among patients with ≤−10° of extension was not significantly different from that of patients with no hyperextension.

Physiologic knee hyperextension is commonly encountered in clinical practice. DeCarlo and Sell^10^ reported normative data for ROM in uninjured high school athletes and found that 95% of male athletes and 98% of female athletes had knee hyperextension (mean, −5° and −6°, respectively). Interestingly, Ramesh et al^26^ reported an increased prevalence of knee hyperextension among patients who underwent ACLR compared with a matched control group without ACL injury. In the present study, the prevalence of knee hyperextension (≤−5°) was high (38.5%). The hyperextension group was characterized by its younger mean age (27.1 ± 10.5 vs 29.1 ± 11.0; *P* < .001) and higher prevalence of female patients (50.0% vs 44.4%; *P* < .001) compared with the no hyperextension group.

Multiple studies have reported increased force and elongation of the ACL during terminal extension.^2,16,18,22,23,34^ Jagodzinski et al^17^ observed greater force and increased impingement of the ACL during passive knee hyperextension versus full passive extension. Accordingly, knee hyperextension may place excessive force on the ACL graft, potentially leading to increased knee laxity and an increased risk of graft failure. However, despite these biomechanical findings, the literature evaluating the clinical outcomes of ACLR among patients with knee hyperextension is scarce and inconsistent. Benner et al^1^ found that knee hyperextension was not associated with knee laxity (KT-1000 arthrometer), subjective knee function (IKDC and Cincinnati Knee Ratings Scale), or graft tearing or failure (STS laxity >5 mm) after ACLR using a BPTB graft. In a large cohort study, the Multicenter ACL Revision Study group^24^ found that knee hyperextension (≤−5°) was an independent predictor of graft failure within 2 years after revision ACLR. However, it should be noted that the difference in failure rate between the hyperextension and nonhyperextension groups was numerically small (3.2% and 2.9%, respectively) and that the determination of graft failure was based on a telephone follow-up considering magnetic resonance imaging findings and/or revision surgery as evidence of graft failure.

Larson et al^20^ evaluated 183 patients who underwent ACLR (46 BPTB autografts, 85 HT autografts, and 52 allografts) and found that a heel height of >5 cm off the table was a predictor of graft failure (graft rupture or KT-1000 STS >5 mm) and poorer subjective outcomes for the entire cohort. However, knee hyperextension measured using a goniometer alone was not a predictor of graft failure. Guimarães et al^12^ recently reported a 5 times higher failure rate among patients with preoperative knee hyperextension (≤−5°) versus patients with no hyperextension after ACLR performed using HT grafts (14.7% and 2.9%, respectively). Graft failure was defined as increased knee laxity (Lachman test, anterior drawer test, or pivot-shift test 2+ or 3+) with associated reports of instability and magnetic resonance imaging evidence of graft rupture. Similarly, Helito et al^14^ reported that patients with >6.5° of knee hyperextension who underwent ACLR using HT autografts were 14.6 times more likely to experience graft rupture. Moreover, the authors reported worse anterior knee laxity (KT-1000 arthrometer) and subjective knee function (IKDC and Lysholm score) in this group of patients versus those with <6.5° of knee hyperextension, although the differences were numerically small (0.5 mm for knee laxity and 5 points for IKDC and Lysholm score). On the basis of these results, one might hypothesize that an HT graft (which consists of only tendons and is characterized by a relatively long tendon-to-bone healing process) may be prone to the deleterious biomechanical effects of knee hyperextension. Accordingly, Guimarães et al and Helito et al recommended avoiding the use of HT grafts in patients with knee hyperextension.

Comparisons between previous studies and the present study are difficult to make because of differences in the surgical techniques, rehabilitation protocols, and investigated outcomes (eg, graft rupture vs revision ACLR). However, the present study (which comprised a very large cohort of patients) showed that knee hyperextension was not associated with postoperative anterior knee laxity, subjective knee function, or the hazard of revision surgery ≤5 years after primary ACLR using HT autografts. The only significant intergroup differences were seen in the KOOS Symptoms subscale at the 1-year follow-up (hyperextension group, 81.4 ± 16.0; no hyperextension group, 80.3 ± 16.5; *P* = .03) and in the Sport/Rec subscale at the 5-year follow-up (hyperextension group, 73.0 ± 25.6; no hyperextension group, 75.7 ± 24.3; *P* = .02). However, these differences were numerically small and not clinically meaningful (<8-10 points).^29,31^ Furthermore, as Benner et al,^1^ we performed a separate analysis of patients with ≤−10° of extension to further select those with a potentially higher risk of graft failure and revision ACLR and found that the hazard of revision ACLR in this group was not significantly different from that of the group with no hyperextension.

The results of this study are clinically relevant. Our findings showed that patients with physiologic preoperative knee hyperextension undergoing ACLR using HT autografts can expect the same results (postoperative anterior knee laxity, subjective knee function, and hazard of revision ACLR) as those with no preoperative knee hyperextension. Therefore, the presence of knee hyperextension alone should not be considered a contraindication for the use of HT autografts in ACLR.

The main strength of this study was the large number of patients included (N = 6104). Therefore, the study had high statistical power, and its results should be considered generalizable. Moreover, this large cohort enabled a subanalysis of the hyperextension group to investigate the hazard of revision ACLR in patients with knee extension ≤−10°. Another strength of this study was that all patients underwent surgery at the same institution and followed a standardized rehabilitation protocol. Finally, we used arthrometric laxity measurements, which are considered more precise than manual laxity measurements.^9,37^

### Limitations

This study had several limitations. First, ROM measurements may be subject to interobserver variability. However, all physical therapists who performed the measurements were experienced, as they assessed knee ROM using a goniometer on a daily basis. In addition, the technique used was standardized, and the study sample was very large. These factors reduced the influence of this possible limitation. Similar considerations can be applied to arthrometric laxity measurements. Second, we were unable to investigate the association between knee hyperextension and rotational laxity. The pivot-shift test was not registered in a standardized manner in our database; therefore, any assessment would have been difficult. Third, KOOS subscale scores were available for 53.9% and 43.2% of patients at the 2- and 5-year follow-ups, respectively. Unfortunately, attrition is a well-known phenomenon in ACL registries.^6,8,19^ However, the 2- and 5-year KOOS subscale scores were available for 3289 and 2637 patients, respectively, and these samples had high statistical power. Fourth, we had no information on the return to sport or postsurgery activity level. Eventual intergroup differences in these variables may have affected the hazard of revision surgery. However, there were no intergroup differences in preinjury Tegner scores, and potential differences in return to sport or postsurgery activity level would likely have affected the study groups similarly without introducing systematic errors. Another limitation was the absence of data on graft rupture. The SNKLR only contains information about revision ACLR. Therefore, intergroup differences in graft rupture cannot be ruled out. Finally, the −5° cutoff used to classify patients with or without knee hyperextension was arbitrary and based on previous studies.^12,24^ However, an established cutoff value for defining knee hyperextension is lacking in the literature.

## Conclusion

Preoperative passive contralateral knee hyperextension (≤−5°) was not associated with postoperative anterior knee laxity, subjective knee function, or revision surgery ≤5 years after ACLR using HT autografts. Therefore, the presence of knee hyperextension alone should not be considered a contraindication per se for the use of HT autografts in ACLR.
